# Integrated Analysis of Polymerase Family Gene Mutations in Acute Myeloid Leukemia: Clinical Features, Prognosis, and Bioinformatics Insights

**DOI:** 10.3390/medicina60121975

**Published:** 2024-12-01

**Authors:** Jianrong Wu, Chaoban Wang, Wenhao Tang, Ju Gao, Xia Guo

**Affiliations:** 1 Department of Pediatric Hematology, West China Second University Hospital, Sichuan University, Chengdu 610017, Chinawcbisababy3303@gmail.com (C.W.);; 2 Key Laboratory of Birth Defects and Related Diseases of Women and Children, Sichuan University, Ministry of Education, Chengdu 610017, China

**Keywords:** AML, prognostic model, POL family genes, bioinformatics analysis

## Abstract

*Background and Objectives*: The long-term prognosis of acute myeloid leukemia (AML) is challenging due to limited understanding of the molecular markers involved in its development. This study investigates the role of DNA polymerases in AML to offer new insights for diagnosis and treatment. *Materials and Methods*: A retrospective study on pediatric AML patients with POL gene family mutations from 2021 to 2024 was conducted. Patients were categorized based on risk stratification criteria, and the DAH regimen was used for induction chemotherapy. Bioinformatics analysis integrated data from various databases to identify key genes and develop survival analysis plots and AUC curves. *Results*: The study included 59 pediatric AML patients, revealing no significant differences in demographic or clinical characteristics between those with and without POL family gene mutations. However, patients with POL gene mutations showed higher complete remission rates after initial DAH chemotherapy (91.67% vs. 59.57%, *p* = 0.03607), indicating a potential treatment benefit. High expression of four POL genes (POLD1, POLE, POLG, and POLQ) in bone marrow and immune cells suggests their crucial role in hematopoiesis and immune response. Survival analysis across different datasets indicated that AML patients with overexpressed POL family genes had significantly worse outcomes, proposing these genes as potential prognostic biomarkers for AML. *Conclusions*: This study on pediatric AML demonstrates that POL gene family mutations are associated with higher remission rates post-chemotherapy, indicating their potential as prognostic markers. Bioinformatics analysis emphasizes the significance of these mutations in AML, highlighting their impact on disease prognosis.

## 1. Introduction

Acute myeloid leukemia (AML) is a clonal malignancy characterized by abnormal hematopoiesis, leading to impaired differentiation and uncontrolled proliferation of immature blasts [1]. Based on morphology–immunology cytogenetics–molecular biology (MICM) identification of abnormal hematopoietic cells in the bone marrow, a relatively comprehensive treatment and follow-up mechanism has been established [2]. However, in pediatric leukemia, unlike acute lymphoblastic leukemia, the long-term prognosis of AML remains a significant challenge [3]. One reason for this is our insufficient understanding of molecular markers involved in the disease’s development and progression.

DNA polymerases are a class of enzymes that catalyze the polymerization of deoxyribonucleotide triphosphates (dNTPs) into progeny DNA, using parental DNA or RNA as templates. Within mammalian cells, there are predominantly five types of DNA polymerases, designated as DNA polymerase alpha (α), beta (β), gamma (γ), delta (δ), and epsilon (ε) [4]. In recent years, an increasing number of studies have found that DNA polymerase is closely related to the occurrence, development, treatment, and prognosis of various tumors. Research on gastrointestinal tumors has revealed that individuals with polymerase ε (POLE) mutations are prone to developing colorectal cancer (CRC) and other gastrointestinal tumors. This mutation is likely an initiating event that upregulates the expression of immune checkpoints. Targeting this process could enhance the efficacy of immune checkpoint inhibitors in treatment [5,6,7,8]. In research on endometrial cancer, POLE mutations account for 7% to 12% of all gene mutations in endometrial cancer. In a study specifically targeting International Federation of Gynecology and Obstetrics (FIGO) stage 3 endometrial cancer, the mutation rate can be as high as 20% [9]. Endometrial cancer patients with POLE mutations have a better prognosis, with significantly lower rates of recurrence and mortality compared to those with wild-type POLE. In high-grade endometrial cancer, patients with POLE mutations rarely experience recurrence, while the recurrence rate in wild-type POLE patients can be as high as 30.9% [9,10,11,12,13]. In bladder cancer research, it has been found that DNA polymerase delta 1 (POLD1) is highly expressed in bladder cancer tissues compared to adjacent tissues. It is also more highly expressed in muscle-invasive bladder cancer than in non-muscle-invasive bladder cancer and is associated with prognosis. Further experiments conducted in vitro and in vivo have demonstrated that POLD1 promotes the proliferation and metastasis of bladder cancer by stabilizing MYC [14,15].

Similarly, DNA polymerase theta (POLQ) is closely related to the occurrence and development of various tumors. Compared to normal tissues, POLQ expression is significantly upregulated in breast cancer tissues, and high expression of POLQ is associated with poor clinical prognosis, with a 4.3-fold increased risk of death in patients with high POLQ expression [16]. Another study on breast cancer has confirmed that high POLQ expression can serve as an independent prognostic factor for breast cancer, indicating a poorer prognosis [17]. Additionally, research has found that POLQ expression in lung adenocarcinoma tissues is higher than in normal tissues [18].

Research on the POL gene family in leukemia treatment is still in its early stages. One study found that POLQ can protect leukemia cells from DNA damage caused by chemotherapy drugs. In addition, the study showed that inhibitors of oncogenic tyrosine kinases (OTK) or DNA–protein crosslink (dpc)-inducing drugs, such as etoposide, enhanced the anti-leukemia effects of POLQ inhibitors both in vitro and in vivo [18]. Furthermore, research has shown that DNA polymerase beta (POLB) exhibits increased activity in chronic myeloid leukemia (CML). During the evolution of CML from the chronic phase to the accelerated phase, an excess of POLB may contribute to the observation of genetic instability [19].

Currently, the diagnosis of AML relies mainly on the MICM classification, with particular emphasis on immunophenotyping and molecular biology examinations. In the past, the establishment of molecular genetics in diagnosis was based on commonly encountered gene mutations, gene fusions, or chromosomal abnormalities that were serendipitously discovered during clinical follow-ups. Due to technological limitations, many potential molecular markers may have gone undetected [20]. Starting from 2021, transcriptome sequencing was applied to AML patients, and in 2022, the World Health Organization updated its diagnostic classification to highlight the critical role of cellular and molecular genetic abnormalities in the diagnosis and treatment guidance of AML [21,22].

Through integrated analysis of whole-transcriptome and -genome sequencing, we have identified a larger number of potential genetic abnormalities. Transcriptome analysis, particularly in acute myeloid leukemia (AML) patients, has provided a more comprehensive dataset, offering novel insights into the molecular mechanisms underlying this disease. However, due to insufficient clinical data reporting, the clinical benefits and potential mechanisms of these newly discovered gene mutations remain unclear.

In this study, we have compiled information on AML patients with DNA polymerase mutations, described their basic clinical characteristics, and utilized public databases to explore the potential role of DNA polymerase in the occurrence and development of AML.

## 2. Methods

### 2.1. Clinical Data Collection

This study was conducted in accordance with the Declaration of Helsinki, and the protocol was approved by the Ethics Committee of West China Second University Hospital (approval code: YXKY2023062; approval date: 10 November 2023). Table of Abbreviations as Table S1.

We conducted a retrospective study on 12 pediatric patients diagnosed with acute myeloid leukemia (AML) carrying mutations in the POL gene family from 1 June 2021 to 1 March 2024. The control group consisted of 47 pediatric AML patients diagnosed between 1 October 2015 and 31 December 2019. Acute promyelocytic leukemia was excluded due to differences in treatment approaches. All patients provided written consent for the use of their clinical data.

Transcriptome data acquisition: Bone marrow samples of 2–3 mL were obtained via posterior iliac crest puncture from patients and sent to Kindstar Global Gene Company (Chengdu, China) for transcriptome sequencing. The specific process is as follows: Using the RiboZero method, ribosomal RNA was removed from the total RNA, followed by reverse transcription into cDNA. cDNA was used as a template to construct libraries compatible with sequencing. RNA from patient samples was analyzed at the whole-transcriptome level using the Illumina NovaSeq 6000 platform, allowing for the analysis of gene fusions and SNV variants at the RNA level. Bioinformatics analysis methods for gene mutations and gene fusions: Sequencing reads were aligned to the Ensembl GRCh37 reference genome using the STAR software v2.7.10a. Variant detection was performed using the varscan software v2.4.6, including mutation threshold filtering, SNV variant discovery, and genotyping. Gene fusion prediction was carried out using arriba v2.0.0. Mutation results were annotated downstream using annovar v.exac03, with annotation databases primarily including ClinVar, dbSNP, 1000Genomes, gnomAD, ExAC, COSMIC, etc. Fusion gene databases included (but were not limited to) COSMIC, FusionCancer, My Cancer Genome, etc.

### 2.2. Diagnostic Criteria [23,24]

All suspected cases must undergo morphology–immunology cytogenetics–molecular biology (MICM) diagnosis and classification, and must meet one of the following criteria:

(1) Bone marrow with primitive or immature granulocytes ≥ 20%.

(2) If bone marrow has <20% primitive immature granulocytes but has specific genetic abnormalities characteristic of primary AML, such as t (8;21), inv (16).

(3) Myeloid sarcoma (extramedullary myeloid tumor, granulocytic sarcoma, or chloroma), regardless of evidence of bone marrow or peripheral blood leukemia cell infiltration, must have evidence of myeloid differentiation. For myeloid sarcoma without infiltration of leukemia cells in bone marrow or peripheral blood, there must be a pathological diagnosis.

### 2.3. Risk Stratification [23,24]

Newly diagnosed patients are classified into low-risk/intermediate-risk/high-risk categories based on examination results, as follows:

(1) Low risk: meeting all four criteria simultaneously: ① having one of the following favorable genetic markers: t(8;21)/AML1-ETO or RUNX1-T1RUNX1, inv(16) or t(16;16)/CBFβ-MYH11, normal karyotype with NPM1 mutation or CEBPα double mutation; ② white blood cell count (WBC) ≤ 100 × 10^9^/L at diagnosis; ③ excluding myeloid sarcoma, central nervous system leukemia, and testicular leukemia; ④ bone marrow minimal residual disease (MRD) < 10^−3^ on Day 28 after the first course of induction therapy, or complete remission in bone marrow (i.e., <5% blast cells) if MRD testing is unavailable.

(2) High risk: having one of the following factors: ① having one of the following unfavorable genetic markers: monosomy 5 or 7, 5q-, 7q-, 12p/t(2;12)/ETV6-HOXD, excluding MLL rearrangements with t(9;11), t(6;9)/DEK-NUP214 or DEK-CAN, t(7;12)/HLXB9-ETV6, t(9;22)/BCR-ABL1, t(16;21)/TLS-ERG or FUS-ERG, complex karyotype (three or more genetic abnormalities excluding favorable karyotype), c-kit mutation, FLT3-ITD mutation, RUNX1 mutation, or TP53 mutation; ② transformed AML: including therapy-related AML, induced post-chemotherapy or radiotherapy AML, a rare type of leukemia related to treatment; AML transformed from myelodysplastic syndromes (MDS); ③ myeloid sarcoma; ④ bone marrow MRD ≥ 10^−2^ on Day 28 after the first course of induction therapy; if MRD testing is unavailable, bone marrow blast cells ≥ 20%.

(3) Intermediate risk: patients were classified as intermediate risk if they exhibited either of the following: ① presence of the [t(8;21) or inv(16) or t(16;16)] translocation along with a c-KIT mutation or ② clinical characteristics that fell between the defined criteria for the low-risk and high-risk groups.

### 2.4. Treatment Protocol [25,26,27]

All newly diagnosed patients receive the DAH regimen as the first course of induction chemotherapy, which includes daunorubicin 40 mg/m^2^ every other day for 3 doses from Day 1 to Day 5; Ara-C, 100 mg/m^2^, twice daily from Day 1 to Day 7; Homoharringtonine, 3 mg/m^2^, once daily from Day 1 to Day 5. Bone marrow remission + MRD assessment is conducted on Day 21 and/or Day 28 of the first course of induction chemotherapy, with Day 28 as the final assessment point. If complete remission is achieved by Day 21, no assessment on Day 28 is needed.

### 2.5. Bioinformatics Analysis

#### 2.5.1. Data Sources and Websites

The following data sources and websites were used: HPA database (Human Protein Atlas) (https://www.proteinatlas.org, accessed on 13 May 2024), DICE database (Database of Immune Cell Expression, Expression quantitative trait loci (eQTLs) and Epigenomics) (https://dice-database.org, accessed on 13 May 2024), String database (https://string-db.org, accessed on 13 May 2024), AlphaFold Protein Structure Database (https://alphafold.ebi.ac.uk, accessed on 13 May 2024), TCGA database (The Cancer Genome Atlas), Target database, GEO database (Gene Expression Omnibus) (GSE37642, https://www.ncbi.nlm.nih.gov/geo/query/acc.cgi?acc=GSE138300, accessed on 13 May 2024), Reactom pathyway database (https://reactome.org, accessed on 13 May 2024), DAVID Pathway Database (The Database for Annotation, Visualization and Integrated Discovery) (https://david.ncifcrf.gov, accessed on 13 May 2024), GSEA database (Gene Set Enrichment Analysis) (https://www.gsea-msigdb.org/gsea/msigdb/human/collections.jsp#C7, accessed on 13 May 2024), and hipot (https://hiplot.com.cn/, accessed on 13 May 2024).

#### 2.5.2. Data Process

Utilizing data from the HPA and DICE databases, a comprehensive understanding of the POL gene family and its impact on immune cells was acquired. Expression profile and survival data from TCGA, Target, and GSE138300 were obtained for conducting survival analysis of the POL gene family, with meta-analysis yielding integrated results from diverse database sources. Through R programming, gene clusters significantly correlated with the POL gene family were identified, followed by enrichment analysis to elucidate pathways potentially influenced by mutations in the POL gene family. Subsequently, 21 key genes were identified through further using Lasso regression, leading to the generation of survival analysis plots and AUC curves.

Detailed descriptions of the databases and R code have been previously disclosed in our published work [28,29,30].

#### 2.5.3. Statistical Analysis

All statistical analyses in this study were conducted using R v4.4.2. The statistical analysis of clinical data utilized the chi-square test. Survival analysis results were computed using the Kaplan–Meier Plotter. Genes related to the POL gene family were analyzed through the Pearson method using R code, and enrichment analysis results were obtained using DAVID. Significance levels were denoted as follows: * for *p* < 0.05, ** for *p* < 0.01, and *** for *p* < 0.001.

## 3. Results

### 3.1. Clinical Characteristics of Pediatric AML

In this study, a total of 59 AML patients were included (12 with mutations in the POL family genes (Table S2) and 47 patients who had not undergone comprehensive transcriptome screening for mutated genes). There were no differences in gender composition, age distribution, immunophenotyping (M0–M7), initial white blood cell levels, or risk group classification (high, low, or medium group) between the two groups. These results suggest that the POL family genes may not manifest phenotypic differences under the current MICM classification. However, it is noteworthy that after the initial induction remission phase with DAH regimen chemotherapy, a significant difference was observed in the complete remission rate upon bone marrow smear re-examination on Day 21 or Day 28 (91.67% vs. 59.57%, *p* = 0.03607, Table 1) between the two groups. This indicates the potential benefits for AML patients carrying mutations in the POL family genes following treatment.

### 3.2. Descriptive Analysis and Prognosis Analysis of the POL Gene Family

**Figure 1:**
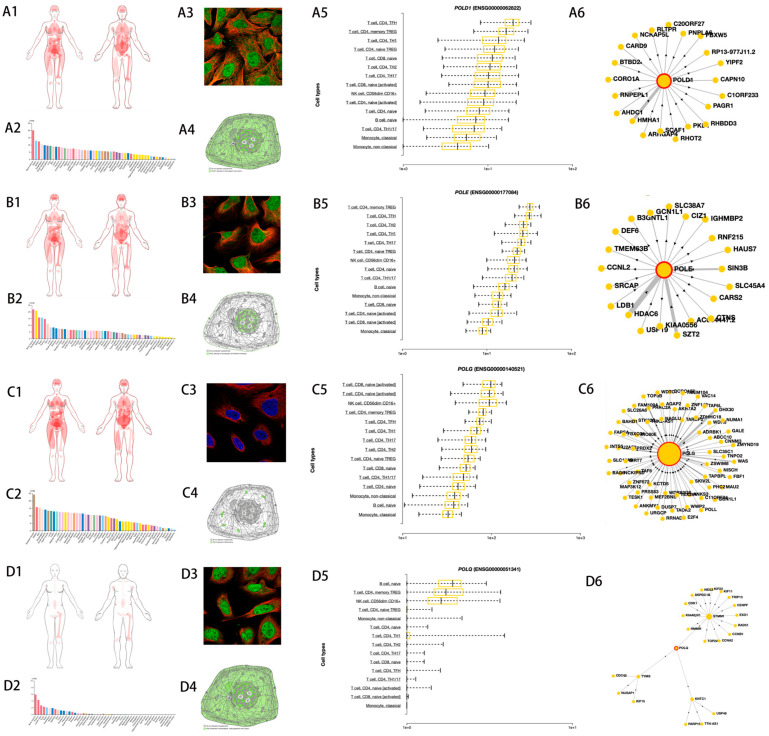
Basic features of the POL gene family. (1) POLD1: (**A1**) human body distribution map; (**A2**) tissue organ expression levels; (**A3**) immunofluorescence localization map of POLD1 (red—cytoplasm; blue—nucleus; green—POLD1); (**A4**) subcellular structure map of POLD1; (**A5**) expression levels of POLD1 in various types of immune cells; (**A6**) network map of POLD1 and associated genes. (2) POLE (**B1**–**B6**). (3) POLG (**C1**–**C6**). (4) POLQ (**D1**–**D6**).

Based on the whole transcriptome results, mutations in four POL family genes, POLD1, POLE, POLG, and POLQ, were detected. Therefore, we primarily analyzed these four genes. Based on the results from the HPA and DICE databases (POLD1: Figure 1(A1–A6); POLE: Figure 1(B1–B6); POLG: Figure 1(C1–C6); POLQ: Figure 1(D1–D6)), all four genes are highly expressed in the bone marrow, which may be related to active hematopoietic function. Subcellular structural results indicate that POLD1 and POLQ are expressed in both the nucleus and cytoplasm, while POLE is mainly expressed in the nucleus, and POLG is primarily expressed in the mitochondria. Furthermore, results from immune cell subgroups suggest that these four genes are highly expressed in different immune cells. Combined with their gene linkage results, these findings suggest that POL family genes may play an important role in various functions of immune cells, providing evidence for the involvement of POL family gene mutations in the occurrence and development of AML.

**Figure 2:**
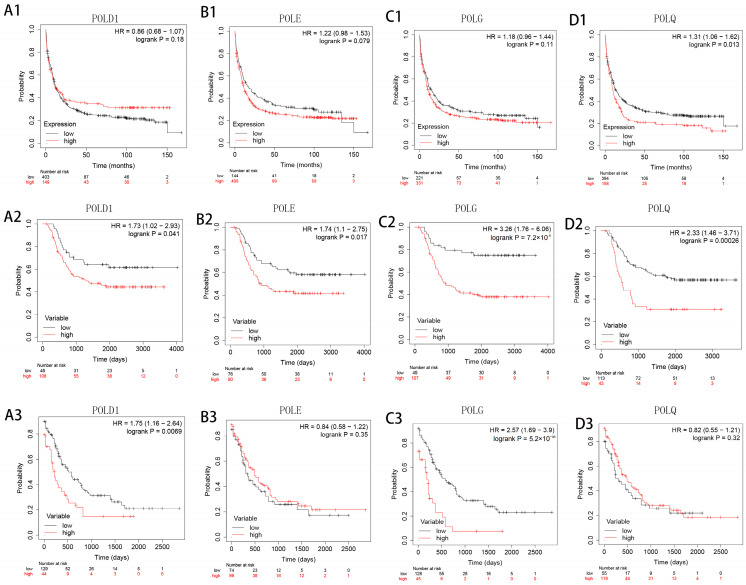
Survival analysis of the POL gene family in the GSE37642, TARGET, and TCGA databases: (1) POLD1 ((**A1**) GSE37642, (**A2**) TARGET, and (**A3**) TCGA); (2) POLE ((**B1**) GSE37642, (**B2**) TARGET, and (**B3**) TCGA); (3) POLG ((**C1**) GSE37642, (**C2**) TARGET, and (**C3**) TCGA); (4) POLQ ((**D1**) GSE37642, (**D2**) TARGET, and (**D3**) TCGA).

Subsequently, we analyzed the impact of POL family genes on survival events in three data sources (GSE37642, n = 552; TARGET database, n = 156; TCGA database, n = 173). The results indicate that in all significant cases (Figure 2(A2,A3,B2,C2,C3,D1,D2)), high expression of POL family genes is associated with poor prognosis, with relative hazards ranging from 1.73 to 3.26. These results suggest that POL family genes may be associated with the long-term prognosis of AML patients and can serve as potential prognostic biomarkers.

### 3.3. Bioinformatics Pathway Analysis and Molecular Biomarker Prognostic Model

**Figure 3:**
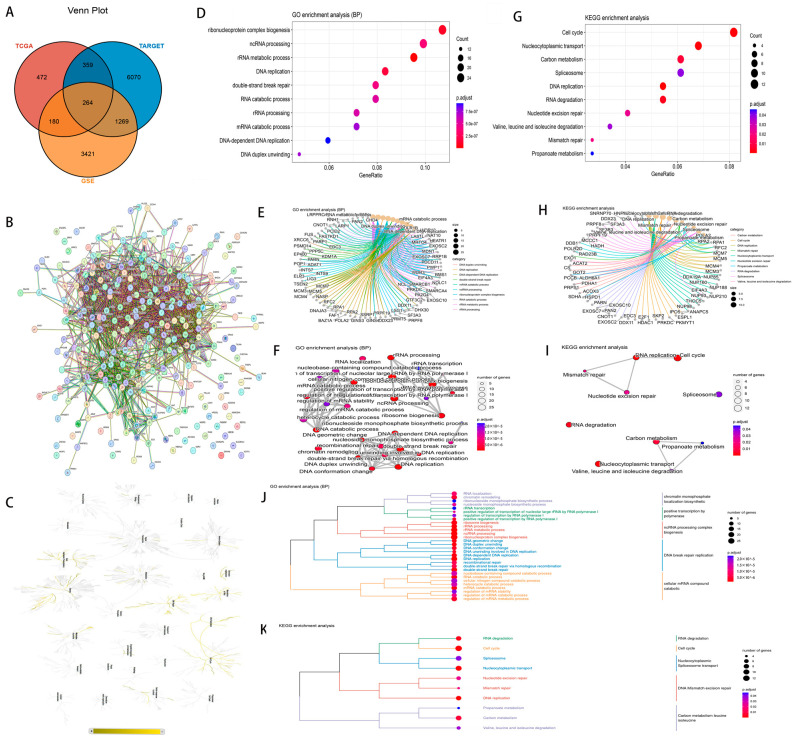
Bioinformatics analysis of the POL gene family. (1) (**A**) Venn diagram, 264 common genes; (2) (**B**) PPI network diagram; (3) (**C**) Reactome enrichment analysis; (4) GOBP enrichment analysis ((**D**) bubble chart, (**E**) pathway-gene chart, (**F**) gene network diagram, and (**J**) dendrogram network diagram); (5) KEGG enrichment analysis ((**G**) bubble chart, (**H**) pathway-gene chart, (**I**) gene network diagram, and (**K**) dendrogram network diagram).

Based on the expression profile data of GSE37642, 5135 genes are significantly associated with the POL gene family. Using TCGA expression profile data, 1275 genes are significantly associated with the POL gene family. Furthermore, based on TARGET expression profile data, 7962 genes are significantly associated with the POL gene family. The Venn diagram indicates a significant correlation of 264 genes with the POL gene family across multiple databases (Figure 3A). The PPI network of these genes was obtained from the String database (Figure 3B). Enrichment analysis results of GOBP, KEGG, and Reactome based on these genes suggest that these enriched genes mainly affect processes such as the cell cycle, and DNA and RNA metabolism (Figure 3C–K).

**Figure 4:**
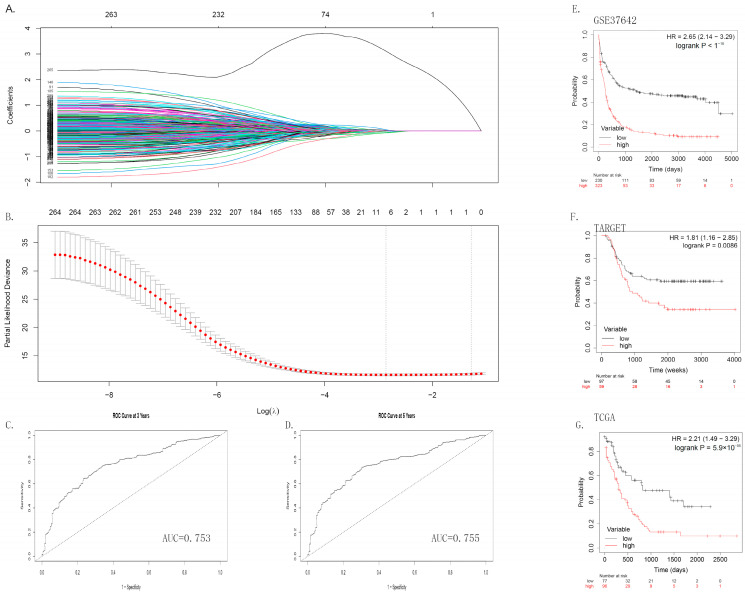
Lasso regression and risk–survival analysis plot. (1) Lasso regression plot (**A**,**B**); (2) AUC curve (3-year expected survival period (**C**), 5-year expected survival period (**D**)); (3) survival curve based on risk value ((**E**) GSE37642, (**F**) TARGET, and (**G**) TCGA).

Subsequently, using GSE37642 data as internal data, Lasso regression was performed (Figure 4A,B), with a final inclusion of 21 genes in the model analysis. The model function is represented as Risk = 0.10422040 × PCID2 + 0.07340848 × EIF4A3 + 0.07215384 × SLC39A14 + 0.03649681 × PDHA1 + 0.03099477 × YARS + 0.01937387 × PLCG2 + 0.01297111 × SDHA + 0.01045514 × SCD + 0.00582637 × C8orf33 − 0.138781901 × RNH1 − 0.107139014 × MTMR4 − 0.067400210 × LARP1 − 0.058022633 × LIG3 − 0.043670449 × PRPF8 − 0.042635170 × E2F1 − 0.037351980 × UXS1 − 0.034852018 × NRF1 − 0.019641953 × POLR3D − 0.017298750 × MLEC − 0.015060838GOLGA3 − 0.002227719 × POLR3. The AUC curve indicates an AUC value of 0.753 for the 3-year survival rate (Figure 4C) and an AUC value of 0.755 for the 5-year survival rate (Figure 4D). Survival analysis based on this risk value suggests a significant association with survival events in AML patients (HR = 2.65 [2.14–3.29], *p* < 1 × 10^−16^, Figure 4E). Furthermore, using TCGA and TARGET databases as external data for validation, the risk value still plays a role in predicting adverse prognosis in TCGA (HR = 1.81 [1.16–2.85], *p* = 0.0086, Figure 4F) and TARGET (HR = 2.21 [1.49–3.29], *p* = 5.9 × 10^−5^, Figure 4G) databases. Based on the above results, mutations in the POL family genes may lead to a downregulation of the expression levels of POL family genes, consequently resulting in a relatively favorable prognosis.

## 4. Discussion

The treatment of childhood AML currently remains primarily based on chemotherapy, which includes induction therapy and consolidation therapy. Although there are various regimens for induction remission, the basic framework still consists of the classic regimen of daunorubicin (DNR) and cytarabine (Ara-C). Daunorubicin exerts its effects by inhibiting topoisomerase II, thereby hindering DNA replication and transcription. Cytarabine is a pyrimidine nucleoside analog that inhibits DNA polymerase, thus blocking DNA synthesis. Homoharringtonine (HHT), an alkaloid derived from the tree Cephalotaxus harringtonia, is a protein synthesis inhibitor and also promotes apoptosis to exert its anti-tumor effects [31]. Under the same treatment regimen, genetic abnormalities in a patient’s leukemia cells may lead to inconsistent responsiveness to chemotherapeutic drugs.

In previous studies, POL family genes have been implicated in the progression of various cancers, such as colon cancer, endometrial cancer, and bladder cancer [5,6,7,8,9,10,11]; however, research on their role in AML has been limited. An important foundation is that abnormalities in POL family genes may lead to inconsistent therapeutic responses to cytarabine, a chemotherapeutic drug that works by affecting DNA polymerase-dependent processes, in leukemia cells.

We explore the intricate relationship between DNA polymerase mutations within the POL gene family and their impact on pediatric acute myeloid leukemia (AML), a formidable malignancy characterized by the aberrant proliferation of immature blood cells. Despite advancements in diagnostic and treatment strategies, the prognosis of pediatric AML remains bleak, highlighting the urgent need for a deeper molecular understanding and identification of novel prognostic markers [32,33].

Our investigation commenced with the enrollment of 59 pediatric AML patients, among which 12 cases exhibited definitive POL family gene mutations. This selection was guided by burgeoning evidence indicating a significant role for DNA polymerases in the development of cancer, attributed to their essential functions in DNA replication and repair. Notably, mutations in these enzymes have been associated with various cancers, including gastrointestinal and endometrial cancers, impacting disease onset, progression, and therapeutic response [9,10,11].

Our methodology was twofold: a detailed clinical analysis to compare demographics, clinical characteristics, and treatment outcomes between the two cohorts, and a rigorous bioinformatics approach utilizing data from public databases such as GSE, TCGA, and TARGET. This allowed us to dissect the expression patterns, subcellular localization, and potential functional implications of POL gene mutations in AML.

Clinically, there were no significant differences in gender distribution, age at diagnosis, or initial white blood cell count between patients with and without POL gene mutations. However, a striking divergence emerged in treatment outcomes. Patients harboring POL gene mutations demonstrated a markedly higher complete remission rate following the initial induction chemotherapy phase compared to their mutation-negative counterparts (91.67% vs. 59.57%, *p* = 0.03607), suggesting a potential therapeutic advantage for this subgroup.

Bioinformatics analyses have significantly deepened our understanding. Mutations were identified in four pivotal POL genes: POLD1, POLE, POLG, and POLQ. These genes were found to have elevated expression levels in the bone marrow, suggesting their critical involvement in hematopoiesis. Furthermore, their expression across various immune cell types indicated a more expansive role in immune regulation. Intriguingly, survival analysis leveraging data from GSE, TCGA, and TARGET datasets demonstrated that AML patients with high expression of POL family genes experienced notably poorer outcomes compared to those without such mutations, highlighting the prognostic significance of these genetic alterations. This implies that the presence of POL family gene mutations might impair the expression levels of POL family genes, thereby exerting a protective effect. The potential underlying mechanisms could involve pathways related to the cell cycle and the metabolism of DNA and RNA.

In our study, although we have unveiled the potential role of DNA polymerase family genes in pediatric acute myeloid leukemia (AML), there are several limitations to our research: (1) Due to the typically longer follow-up periods required for pediatric AML patients compared to adults, our single-center study is currently unable to provide comprehensive long-term prognostic data. Thus, a longer follow-up period is necessary to obtain more complete prognostic information, which is crucial for understanding the impact of POL family gene mutations on the prognosis of pediatric AML patients. (2) Our study relies on bioinformatics analysis to explore the role of the POL gene family in AML but lacks further experimental validation. Although this is the first analysis of the potential impact of POL family genes in AML research and we have proposed a prognostic model for risk assessment, the accuracy of bioinformatics results may be questioned due to the absence of experimental data. This highlights the importance of future laboratory validation work to ensure that our findings are biologically reliable. (3) Our study is based on a relatively small cohort, which may limit the generalizability and statistical power of our results. (4) We acknowledge the limitation regarding the control group. Although the control group of 47 patients did not undergo comprehensive transcriptome screening for mutated genes, making it impossible to confirm whether they have mutations in the polymerase genes, we believe that the study still provides valuable insights. Our study suggests that patients with POL gene mutations show different treatment responses compared to conventional AML patients.

In summary, our study, based on single-center and public database analyses, suggests that POL family gene mutations may be associated with higher remission rates post-chemotherapy, indicating their potential as prognostic markers. For patients with POL family gene mutations, alternative therapies targeting DNA polymerase for resistant cancers hold important promise. These findings pave the way for future research into targeted therapies that could exploit these molecular vulnerabilities, offering hope for improved management strategies in this challenging pediatric malignancy.

## Figures and Tables

**Table 1:** Patient clinical characteristics between the POL family genes positive and control group.

POL Gene Positive Group (n = 12)			Control Group (n = 47)			*p* Value
	**N**	**%**		**N**	**%**	
**Gender**			**Gender**			
Female	6	50.00%	Female	20	42.55%	0.64288
Male	6	50.00%	Male	27	57.45%	
**FAB classification**			**FAB classification**			0.36768
M0	0	0.00%	M0	0	0.00%	
M1	0	0.00%	M1	0	0.00%	
M2	4	33.33%	M2	29	61.70%	
M4	4	33.33%	M4	8	17.02%	
M5	3	25.00%	M5	8	17.02%	
M6	0	0.00%	M6	0	0.00%	
M7	1	8.33%	M7	1	2.13%	
Unknown	0	0.00%	Unknown	1	2.13%	
**AGE**			**AGE**			0.45521
<3	4	33.33%	<3	8	17.02%	
3–10	4	33.33%	3–10	19	40.43%	
10–15	4	33.33%	10–15	20	42.55%	
15–18	0	0.00%	15–18	0	0.00%	
**Risk group**			**Risk group**			0.10342
LR	2	16.67%	LR	11	23.40%	
MR	2	16.67%	MR	17	36.17%	
HR	8	66.67%	HR	19	40.43%	
**Reached CR after cycle 1 of induction**			**Reached CR after cycle 1 of induction**			0.03607 *
Yes	11	91.67%	Yes	28	59.57%	
No	1	8.33%	No	19	40.43%	

* for *p* < 0.05.

## Data Availability

The data that support the findings of this study are available from the corresponding author upon reasonable request.

## Supplementary Materials

The following supporting information can be downloaded at: https://www.mdpi.com/article/10.3390/medicina60121975/s1, Table S1: Table of Abbreviations. Table S2: Information on POL Family Gene Variants.
