# Supplementary material for: Integrated Analysis of Polymerase Family Gene Mutations in Acute Myeloid Leukemia: Clinical Features, Prognosis, and Bioinformatics Insights
Source: Medicina (Kaunas). 2024 Dec 1;60(12):1975. doi: 10.3390/medicina60121975 (PMC11676477; doi:10.3390/medicina60121975)
Supplement: Supplementary file 1 [file medicina-60-01975-s001.zip › TableS1.docx]

**TableS1.** Table of Abbreviations

| Abbreviation | Full Expression |
| --- | --- |
| POL | Polymerase |
| AML | Acute Myeloid Leukemia |
| POLE | Polymerase ε |
| MICM | morphology-immunology cytogenetics-molecular biology |
| FIGO | International Federation of Gynecology and Obstetrics |
| POLD1 | DNA polymerase delta 1 |
| POLQ | DNA polymerase theta |
| OTK | oncogenic tyrosine kinases |
| POLB | DNA polymerase beta |
| CML | chronic myeloid leukemia |
| MRD | bone marrow minimal residual disease |
| MDS | myelodysplastic syndromes |
| DAH | Daunorubicin，Ara-C (cytarabine)，Homoharringtonine |
